# Desensitization Protocol to Carboplatin: A Technical Report

**DOI:** 10.7759/cureus.24515

**Published:** 2022-04-26

**Authors:** Cornelia Nitipir, Andreea Parosanu, Ana Maria Popa, Horia Cotan, Mihaela Olaru, Cristian Iaciu, Cristina Orlov Slavu

**Affiliations:** 1 Oncology, Elias Emergency University Hospital, Bucharest, ROU; 2 Oncology, Carol Davila University of Medicine and Pharmacy, Bucharest, ROU

**Keywords:** reaction, protocol, hypersensitivity, desensitization, carboplatin

## Abstract

Carboplatin is one of the most widely used chemotherapy agents for solid tumors. One of the most common major side effects is the hypersensitivity reaction, and the likelihood of it increases with the number of courses given. Permanent discontinuation of carboplatin is not required if this side effect occurs. Using an effective desensitization protocol, this type of event can be avoided so that the patient continues to benefit from the maximum antitumor effect. This technical report is meant to detail the desensitization protocol designed and followed in the Oncology Clinic of the University Emergency Hospital, Bucharest.

## Introduction

Hypersensitivity reactions occur to carboplatin, a frequently used chemotherapy agent in solid tumor oncology, in up to 30% of cases [1]. The probability of this happening is directly proportional to the number of administrations as follows: 1% in patients who received less than six carboplatin administrations, 27% in patients with a number of administrations between seven and 14, and 46% in patients with more than 15 infusions [2]. If this happens, in many cases, the patient abandons carboplatin and receives a later line of treatment with less benefit. To be able to continue the administration of carboplatin in case of an allergic reaction, desensitization regimes are necessary. The way they are designed can decisively influence the tolerance of patients. This technical report aims to detail the carboplatin desensitization protocol used in the Oncology Clinic of the Elias University Emergency Hospital, Bucharest, Romania.

## Technical report

Desensitization protocol to carboplatin

How to Select Patients for Desensitization Regimen

Patients who will receive a desensitization regimen in our clinic are those who have a positive skin test (the test is performed in all patients with no history of hypersensitivity reactions to carboplatin, except those who had more than six administrations) or those who have had any type of hypersensitivity reaction during or after infusion. The skin test is performed as an intradermal injection, using carboplatin at a concentration of 5 mg/ml. In case of hypersensitivity reactions, all the clinical details of the manifestation are recorded in the medical history, and an allergology consultation is requested. This medical specialty is the one that ultimately decides who is a candidate for the desensitization protocol, and the case is always discussed in the multidisciplinary commission of the hospital. The present regimen is administered only in continuous hospitalization where the patient can be treated together with an intensive-care specialist. The transfer of the patient will be done rapidly in the intensive care unit if the need arises.

Steps in administration

The carboplatin dose was calculated for each administration using the desired Calvert formula below for area under the curve (AUC):

Dose (mg) = AUC (mg/ml/min) x [GFR (ml/min) + 25 (ml/min)].

The glomerular filtration rate (GFR) values ​​were estimated using the Cockcroft-Gault formulas below:

Estimated GFR (male) = ([140-age] × weight in kg)/(serum creatinine × 72)

Estimated GFR (female) = estimated GFR (male) × 0.85.

All patients were given premedication the night before and in the morning of chemotherapy with histamine 2 (H2) receptor antagonist orally or intravenously, dexamethasone 8 mg bid, loratadine 10 mg bid, 5-HT3 serotonin receptor antagonist (granisetron) 3 mg intravenously, and normal saline 1000 ml intravenously. Then, the whole dose of carboplatin is divided into four parts as follows: The first part contains 0.1%, the second part contains 1%, the third part contains 10%, and the last or fourth part contains the rest. Each dose is dissolved in 250 ml of 5% glucose solution.

The protocol starts with the administration of the first and the second doses in a one-hour gap. The administration rate starts with 2 ml per hour (for 15 minutes), followed by 5 ml/hour (for 15 minutes), then 10 ml/hour, and 20 ml per hour (also in 15 minutes). The 10% of the dose is administered in 75 minutes as follows: It starts with 2 ml/hour, then 5 ml/hour, then 10 ml/hour, and finally the concentration of 10 ml/hour will be infused in 30 minutes. The administration time of the remaining dose is a variable. The 2 ml/hour, 5 ml/hour, and 10 ml/hour rates are administered in 15 minutes, and when the 20 ml/hour flow rate is reached, the infusion will continue until the full dose is received. The administration schedule ends with 5% glucose 500 ml IV flush, Ringer 500 ml flush, normal saline 1000 ml IV flush, histamine H2 receptor antagonist (famotidine) 40 mg x 2 bid (at 21:00 pm), magnesium hydroxide 400 mg x 3 bid, and metoclopramide 10 mg x 3 bid. All this information is summarized in Table 1. This schedule was printed and completed with the patients' particular information for each administration.

**Table 1:** Desensitization protocol to carboplatin Bid: Twice daily; 5-HT_3_: 5-hydroxytryptamine.

Day 1				
Personal medication + Premedications prior to desensitization protocol				
H2-receptor antagonist famotidine: 20 mg IV bid				
Dexamethasone: 8 mg IV x 2				
Antihistamine (loratadin): 10 mg bid				
5-HT_3_ Serotonin receptor antagonist (Granisetron): 3 mg IV 20 min every 8 h				
Normal saline: 1000 ml IV				
Carboplatin + 5% glucose 250 mL infuse 1 h	Carboplatin (concentration)	Dilution	Rate (mL/h)	Administration time (60 min)
	1/1000 (0.1%)	5% glucose 250 mL	2 ml/h	15
			5 ml/h	15
			10 ml/h	15
			20 ml/h	15
Carboplatin + 5% glucose 250 mL infuse 1 h	Carboplatin (concentration)	Dilution	Rate (mL/h)	Administration time (60 min)
	1/100 (1%)	5% glucose 250 mL IV	2 ml/h	15
			5 ml/h	15
			10 ml/h	15
			20 ml/h	15
Carboplatin + 5% glucose 250 mL infuse over 1 h	Carboplatin (concentration)	Dilution	Rate (mL/h)	Administration time (75 min)
	1/10 (10%)	5% glucose 250 mL	2 ml/h	15
			5 ml/h	15
			10 ml/h	15
			20 ml/h	30
Carboplatin + 5% glucose 250 mL infuse over 60 min	Carboplatin (concentration)	Dilution	Rate (mL/h)	Administration time (75 min)
	Remainder	5% glucose 250 mL IV	2 ml/h	15
			5 ml/h	15
			10 ml/h	15
			20 ml/h	Until the total dose is administered
5% glucose: 500 mL IV flush				
Ringer: 500 mL flush				
Normal saline: 1000 mL IV flush				
Histamine H_2_ receptor antagonist (famotidine): 40 mg x 2 bid (at 21:00 pm)				
Magnesium hydroxide: 400 mg x 3 bid				
Metoclopramide: 10 mg x 3 bid				
Day 2				
Personal medication + 1, 2, 3, 4, 5, 11, 12, 13, 14, 15				

## Discussion

The mechanism of occurrence of hypersensitivity reactions to carboplatin has two components: the type I hypersensitivity reaction in which immunoglobulin E activates basophils and mast cells and results in the secretion of histamine, prostaglandins, and leukotrienes that will produce anaphylactoid reactions, that is, hypersensitive type II reaction (second stage). Desensitization is based on the principle that exposure to low doses of allergen induces inhibitory mechanisms in mast cells and basophils, and the above sequence is blocked. Moreover, according to the same principle, if hypersensitivity reactions occur during desensitization, they have a lower clinical resonance [3-6].

Patient selection using the carboplatin skin test before treatment is known to be an effective way, with a high negative predictive value of up to 99%. In the case of our clinic, starting the skin test with the seventh administration of carboplatin was preferred because the probability of allergy after this administration increases greatly [7]. Carboplatin skin testing is an essential element in the prophylaxis of hypersensitivity reactions. The concentration used in the intradermal method affects the test result. Obviously, the higher it is, the lower the false-negative rate, but skin necrosis has been reported at concentrations above 10 mg/ml. Hence, 5 mg/ml is used in our protocol, with the injection procedure being a standard one and the injecting staff being trained to do this by an allergist [8].

The duration of administration of the desensitization scheme is perhaps the most important aspect related to this treatment. The longer the duration of administration, the shorter the chance of an allergic reaction. However, regimens with a long time of infusion lead to low patient compliance and the need for longer hospitalization at higher costs. Among the shortest administration times described in the literature, we mention the 3.5-hour protocol used for 129 patients in the research of Altwerger et al., this being the most exhaustive research with this protocol. Patients were included in this trial if they had a positive skin test or if they had a history of hypersensitivity reactions to carboplatin. Another interesting finding in this publication is the link between age and hypersensitivity reactions during the desensitization protocol. Hidden cardiovascular comorbidities must be taken into account in all elderly patients. If the patient presents such comorbidities, the administration of large volumes intravenously can be unsafe. This is also why in our clinic, all patients who will receive desensitization regimens are also evaluated by the cardiologist. The evaluation includes echocardiography with the estimation of the left ventricle ejection fraction (LVEF) [9].

Increasing the dose of the allergen incrementally is the most effective way to avoid an adverse reaction. As our protocol is conceived, it is much easier technically to administer carboplatin in a sequence of four doses by increasing the concentrations 10 times in the first three doses and administering the rest of the amount in the last one, and using the incremental increase of 2, 5, 10, 20 ml/hour [10]. A lower carboplatin infusion rate could minimize the antitumor effect due to the fact that when the exposure time of the drug is longer, the serum drug concentrations are lower. However, it is known that this effect is directly proportional to the carboplatin AUC and not to the peak serum concentration, and this does not change with desensitization regimens [11].

A recent retrospective study intended to verify if desensitization regimens to carboplatin have similar oncological results as classical administration schedules. When 17 patients who received desensitization were compared to a control group of 41 patients, no differences were found between the time to progression, 12-month survival, or overall survival. The same study demonstrated that adverse reactions were also similar between the two groups [12].

## Conclusions

To conclude, the carboplatin desensitization protocols create the possibility to continue the administration of this important chemotherapy agent, even in the presence of past hypersensitivity reactions. These regimens are neither less efficient nor more toxic than classical administration. The technical report of the protocol used in our clinic clearly presents the steps that should be taken to balance the safety and duration of infusion. This article is the first step toward more profound research and a prospective trial including patients from our institution that can prove the clinical benefit of our desensitization regimen.
